# Features of Primary Chronic Headache in Children and Adolescents and Validity of Ichd 3 Criteria

**DOI:** 10.3389/fneur.2019.00092

**Published:** 2019-02-15

**Authors:** Laura Papetti, Irene Salfa, Barbara Battan, Romina Moavero, Cristiano Termine, Beatrice Bartoli, Francesca Di Nunzio, Samuela Tarantino, Pierfrancesco Alaimo Di Loro, Federico Vigevano, Massimiliano Valeriani

**Affiliations:** ^1^Headache Center, Department of Neuroscience, Bambino Gesù Children Hospital, Rome, Italy; ^2^Child Neurology Unit, Systems Medicine Department, Tor Vergata University Hospital of Rome, Rome, Italy; ^3^Child Neuropsychiatry Unit, Department of Clinical and Experimental Medicine, University of Insubria, Varese, Italy; ^4^Child Neuropsychiatry Unit, ASST dei Sette Laghi, Varese, Italy; ^5^Department of Statistical Sciences, Sapienza University of Rome, Rome, Italy; ^6^Center for Sensory-Motor Interaction, Aalborg University, Aalborg, Denmark

**Keywords:** chronic headache, children, chronic migraine, tension-type headache, medication overuse headache, prophylactic therapy

## Abstract

**Introduction:** Chronic headaches are not a rare condition in children and adolescents with negative effects on their quality of life. Our aims were to investigate the clinical features of chronic headache and usefulness of the International Classification of Headache Disorders 3rd edition (ICHD 3) criteria for the diagnosis in a cohort of pediatric patients.

**Methods:** We retrospectively reviewed the charts of patients attending the Headache Center of Bambino Gesù Children and Insubria University Hospital during the 2010–2016 time interval. Statistical analysis was conducted to study possible correlations between: (a) chronic primary headache (CPH) and demographic data (age and sex), (b) CPH and headache qualitative features, (c) CPH and risk of medication overuse headache (MOH), and (d) CPH and response to prophylactic therapies. Moreover, we compared the diagnosis obtained by ICHD 3 vs. ICHD 2 criteria

**Results:** We included 377 patients with CPH (66.4% females, 33.6% males, under 18 years of age). CPH was less frequent under 6 years of age (0.8%; *p* < 0.05) and there was no correlation between age/sex and different CPH types. The risk to develop MOH was higher after 15 years of age (*p* < 0.05). When we compared the diagnosis obtained by ICHD 2 and ICHD 3 criteria we found a significant difference for the undefined diagnosis (2.6% vs. 7.9%; *p* < 0.05), while the diagnosis of probable chronic migraine was only possible by using the ICHD2 criteria (11.9% of patients; *p* < 0.05). The main criterion which was not satisfied for a definitive diagnosis was the duration of the attacks less than 2 h (70% of patients younger than 6 years; *p* < 0.005). Amitriptyline and topiramate were the most effective drugs (*p* < 0.05), although no significant difference was found between them (*p* > 0.05).

**Conclusion:** The ICHD 3 criteria show limitations when applied to children under 6 years of age. The risk of developing MOH increases with age. Although our “real word” study shows that amitriptyline and topiramate are the most effective drugs regardless of the CPH type, the lack of placebo-controlled data and the limited follow-up results did not allow us to conclude about the drug efficacy.

## Introduction

Chronic primary headaches (CPH) are a disabling disorder for children, adolescents, and adults, with a reported prevalence of 2% in adults and 0.78% in adolescents, while the prevalence rises up to 1.75% when including the MOH (1). Nearly 69% of children and adolescents who present to headache specialty clinics have chronic migraine (1). In adolescents and children suffering from this condition, attacks may interfere with the predictability of normal life activities and affect the ability to work, perform routine course and school activities, and maintain functional social relations. CPH determines a huge decrease of the quality of life (1).

Chronic migraine (CM), chronic tension-type headache (CTTH) and new daily persistent headache (NDPH) are classified as CPH in the International Classification of Headache Disorders 3rd edition (ICHD 3). Medication-overuse headache (MOH) is classified among secondary headaches, but it generally affects patients with a pre-existing primary headache. The least common denominator of all these forms of CPH is the persistence of the symptoms for at least 3 months, while the clinical features can vary (2).

CPH may be improved by non-pharmacological treatment, such as lifestyle modifications and complementary therapies (i.e., cognitive behavioral therapy), and/or pharmacological prophylaxis (3).

There are few data concerning the characterization of CPH in the pediatric population, so that most our knowledge emerges from the experience in adulthood. The latest version of the International Classification (ICHD 3) does not include notes for the diagnosis of CPH in pediatric age, although CPH is reported as an increasing condition in children and adolescents with distinct clinical features compared to the adult population (4).

The aims of our “real word” study were: (1) to describe the features of chronic headache in children, and (2) to compare the diagnostic usefulness of ICHD 2 (5) and ICHD 3 criteria. As a secondary aim, we will describe retrospective data of efficacy of the commonly used prophylactic pharmacological therapies.

## Methods

**Figure 1:**
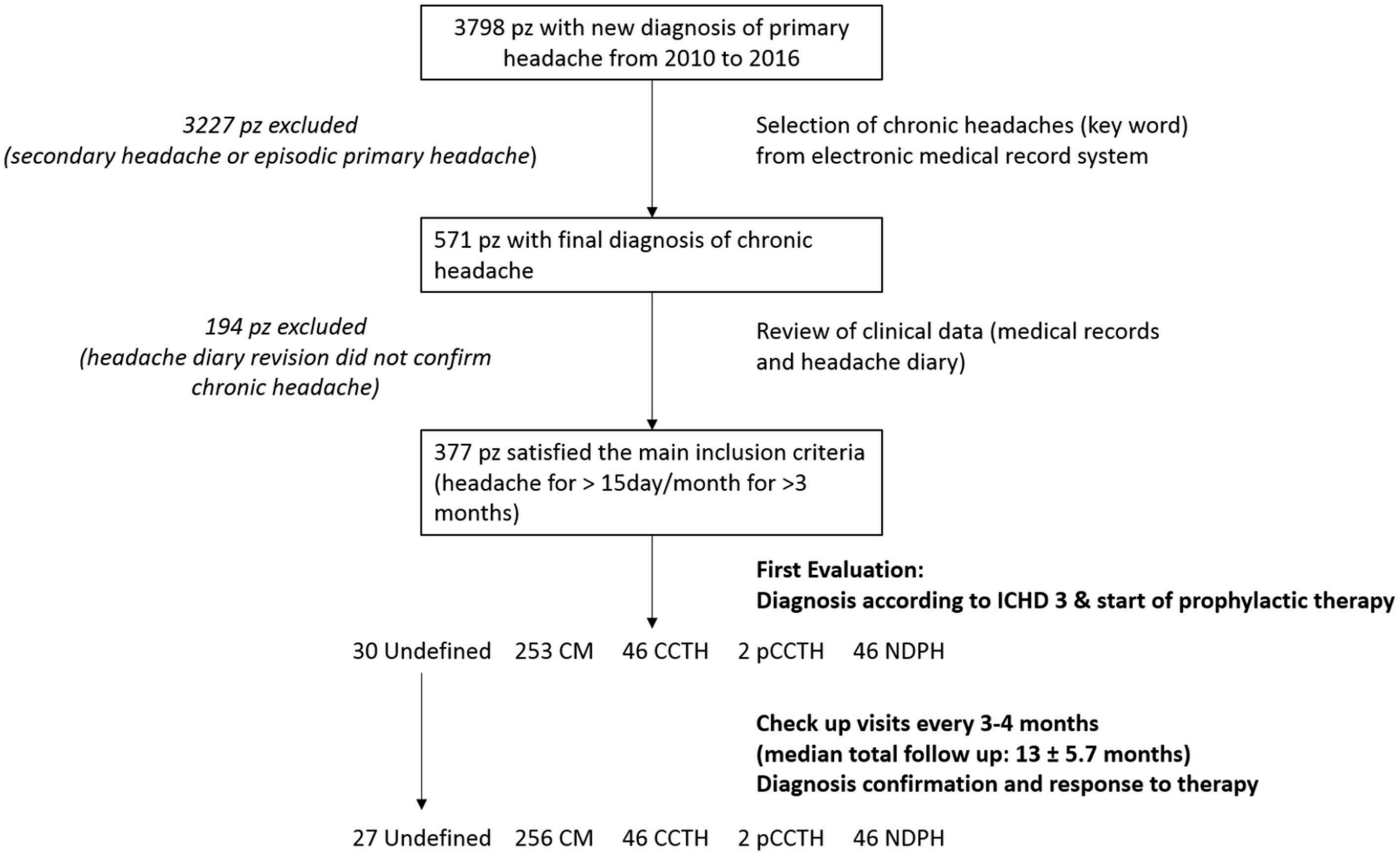
Study methods design.

We retrospectively reviewed the charts of patients attending to the tertiary, university-affiliated, pediatric medical Headache Centers of Bambino Gesù Children and Insubria University Hospital. The design of the study is resumed in Figure 1. The electronic database of the headache clinics was searched for all children and adolescents up to 18 years of age, diagnosed with CPH during the 2010–2016 time interval. Moreover, in CPH population a history of drug overuse supporting the diagnosis of MOH was looked for. The diagnosis was re-evaluated in all cases by using the ICHD-III criteria (2). The main inclusion criteria was history of headache occurring on 15 or more days/month for more than 3 months. Exclusion criteria were headache types other than CPH and the presence of other internist and/or neurological illness. We considered the following CPH types: CM, CCTH, and NDPH. Data on demographics, headache symptoms, and other clinical headache-related parameters were collected from the medical files of the patients who were found eligible to be included in the study. Electronic medical records included the following information: demographic data (age, sex), familiar medical history including headaches, pregnancy and birth history, past medical history, anthropometrical data (weight and height), general physical exam and neurological exam including fundus oculi. Medical charts included also results of possible neuroimaging exams and the data from headache diary. Headache diary reports the number of the attacks for months, duration of the attacks, qualitative features of pain, presence of associated symptoms (nausea, vomiting, phonophobia, and photophobia), intensity of pain, name of drug for the attack and response to therapy for the attack.

Patients were divided into four age groups: 0–6, 7–10, 11–15, and 15–18 years.

Clinical data, concerning duration, qualitative features of the headache attacks, related symptoms and prophylactic pharmacologic therapies were issued from the first and follow up visits. These data were collected from interviews to children and/or their parents. For very young children headache frequency and symptoms were determined by the child's complaints and the parents' impression from the child's behavior (according to the ICHD-III criteria) (2). In addition, parents were questioned about possible medication overuse of their child. The medical interview was always followed by a complete full physical and neurological examination of the patient.

Statistical analysis was conducted by SPPS version 22.0 and χ^2^ test was used to verify possible correlations between: (a) CPH subtypes and population features (age and sex), (b) CPH subtypes and headache qualitative features (nausea, vomiting, phonophobia, and photophobia), (c) predictors of development MOH, and (d) CPH and response to prophylactic therapies (amitriptyline, topiramate, flunarizine, and L-5 hydroxytryptophan). In addition, we performed a comparison between ICHD 2 and ICHD 3 criteria for the diagnosis. A *p*-value of ≤0.05 was considered significant.

Written informed consent was obtained from the parents of the participants in this study. The study was approved by the Ethical Committee of Bambino Gesù Children Hospital.

## Results

### Descriptive Analysis of Chronic Headache Characteristics and Clinical Correlations

We included 377 patients who experienced chronic headache (66.4% females, 33.6% males; *p* > 0.05). Mean age of selected patients was 10.8 years ± 2.5 standard deviation (SD) (range 3.20–18 years). Pain quality, intensity and frequency of the attacks, and associated symptoms are shown in Table 1.

**Table 1:** Headache characteristics in our sample.

**AGE OF PATIENTS**	
0–6 years	31/377 (8.2%)
7–10 years	144/377 (38.2%)
11–14 years	151/377 (40.1%)
15–18 years	51/377 (13.5%)
**HEADACHE TYPES (ICHD 3)**	
Chronic Migraine (CM)	253/377 (67.1%)
Chronic Tensive Type Headache (CTTH)	46/377 (12.2%)
Probable CTTH	2/377 (0.5%)
New Daily Persistent Headache (NDPH)	46/377 (12.2%)
Medication Overuse Headache (MOH)	41/377 (10.8%)
Undefined	30/377 (7.9%)
**PAIN QUALITY**	
Throbbing	94/377 (24.9%)
Gravative	113/377 (29.9%)
Pressing	61/377 (16.1%)
Other qualities	109/377(28.9%)
**PAIN INTENSITY**	
Mild	75/377 (19.8%)
Moderate	132/377 (35%)
Severe	170/377 (45%)
**ATTACK DURATION**	
Less than 1 h	122/377 (32.3%)
Between 1 and 2 h	150/377 (39.7%)
More than 2 h	105/377 (27.8%)
**ASSOCIATED SYMPTOMS**	
Photophobia	225/377 (59.6%)
Phonophobia	258/377 (68.4%)
Nausea and/or vomiting	172/377 (45.6%)

CPH was less frequent under 6 years of age (0.8%; *p* < 0.05), while a significant higher prevalence of CPH was found in females than in males in the age group between 0 and 6 years (23/31 females, 8/31 males) and between 15 and 18 years (41/51 females, 10/51 males) (*p* < 0.05). No significant statistical correlation between age/sex and different CPH types was found. Nausea and vomiting were the two most frequent vegetative symptoms under 10 years of age (*p* < 0.05) while photo/phonophobia were more frequent in patients older than 15 years (*p* < 0.05).

**Figure 2:**
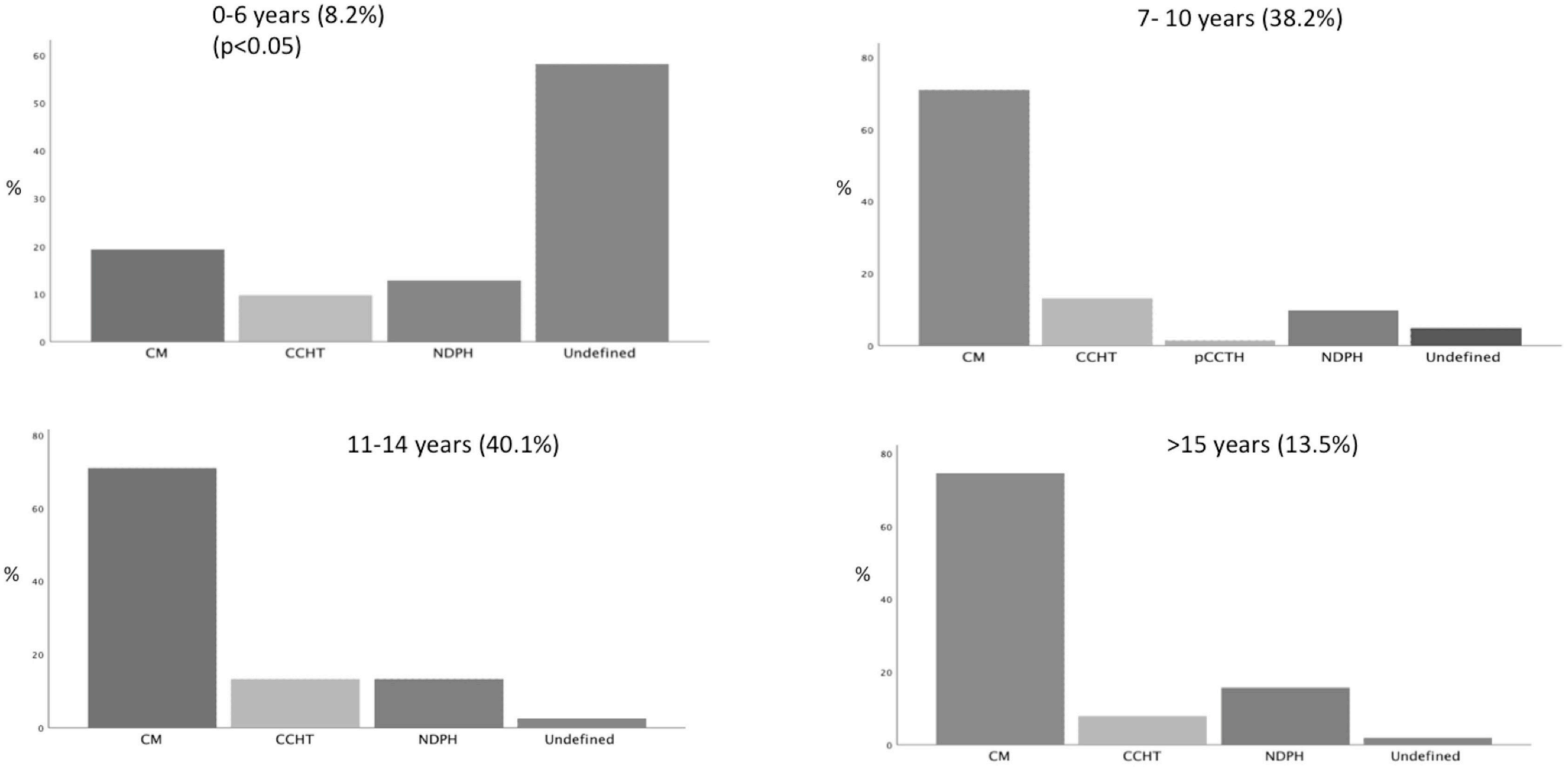
Distributions of headache subtypes (ICHD 3) in relation with age.

As for attack duration, three groups of patients were identified: (1) attack duration was shorter than 1 h in 122 patients (32.3%), (2) it ranged between 1 and 2 h in 150 patients (39.7%), and (3) it was longer than 2 h in 105 patients (27.8%). When the different age-based groups of patients were considered, a significant different distribution of the attack duration was found. In particular, we found that an attack duration shorter than 2 h was more frequent in the patients between 0 and 6 year (70%) as compared to other groups (39.5% in patients between 7 and 10 years, 24.5% in patients between 11 and 14 years and 13.7% in patients older than 14 years) (*p* < 0.05). As consequence of this phenomenon we detected that the distribution of CH subtypes tends to overlap in the age groups between 7 and 10 years, 11–14 years and above 15 years while in patients younger than 6 years, we have a significant increase in the frequency of probable or undefined diagnoses (*p* < 0.05) (Figure 2). The most frequent parameter that did not fill the criteria for a definitive diagnosis in patients under 6 age, was the duration of the attack less than 2 h.

**Figure 3:**
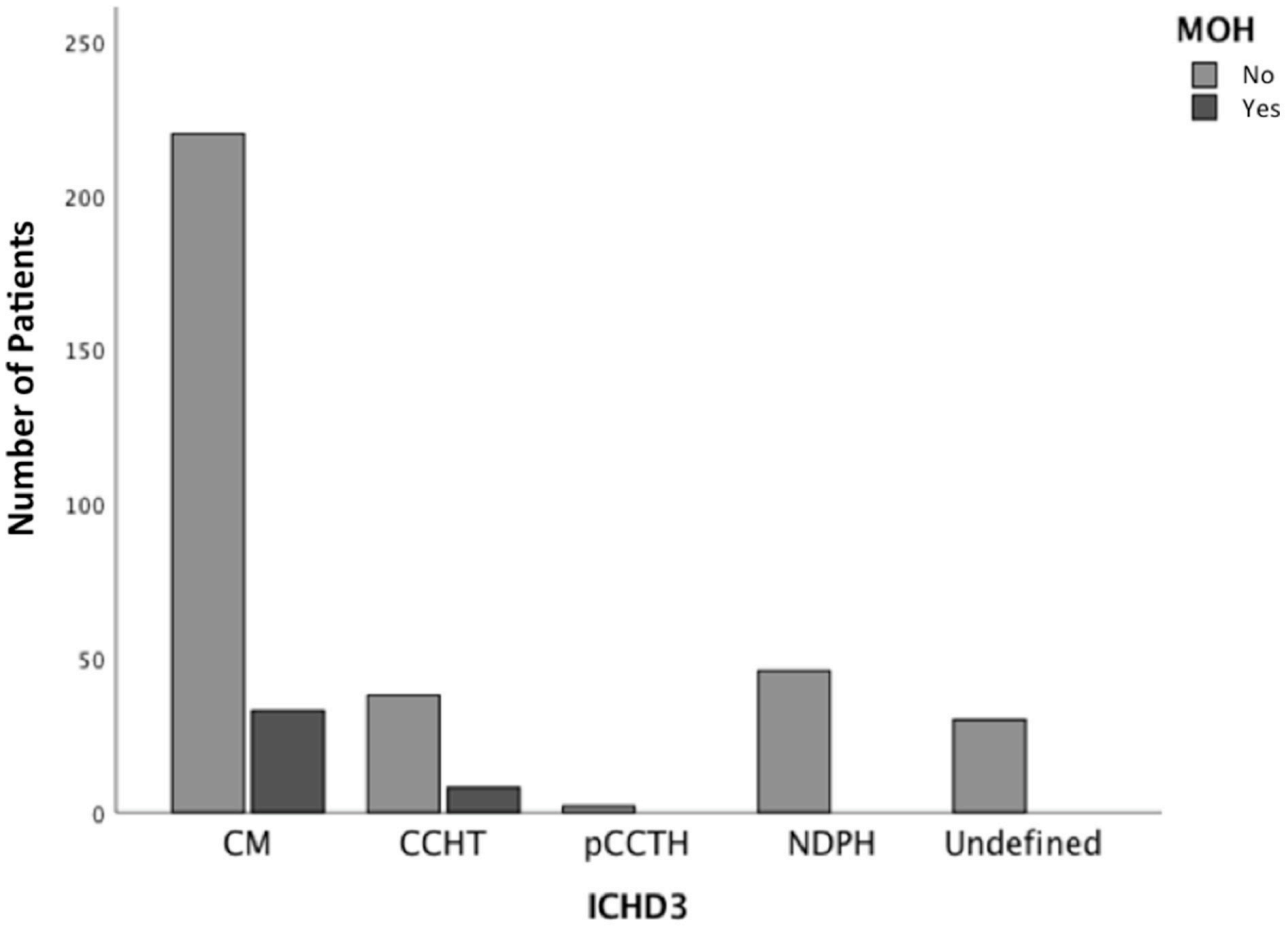
Number of patients with MOH according to headache subtypes.

**Figure 4:**
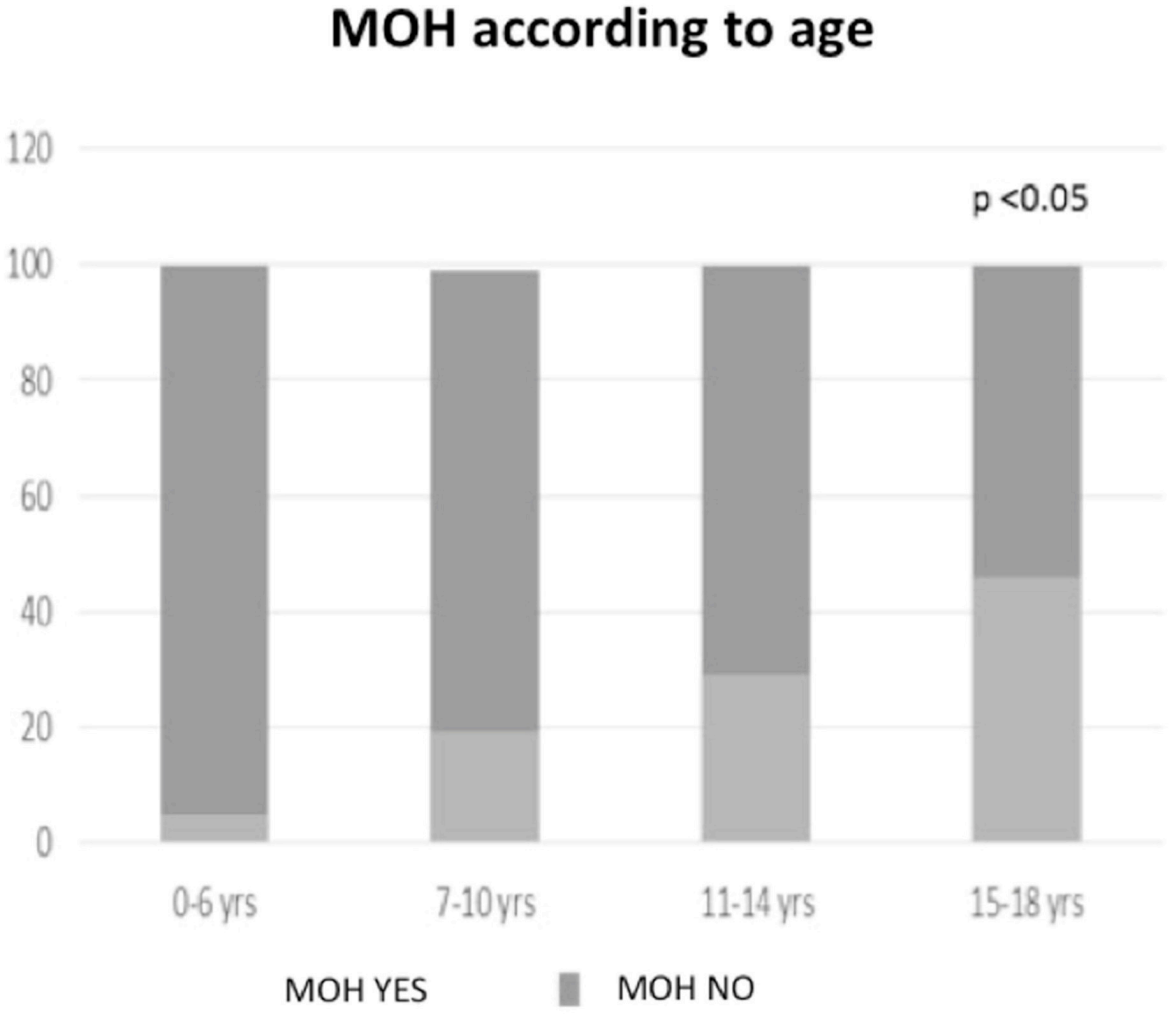
Number of patients with MOH according to patient's age.

MOH was found in 10.8% of patients and interested only patients with CM and CTTH (Figure 3). Ibuprofen was the most frequently overused drug. Excluding the overuse of drugs for the attack, we found that the only clinical factor associated with higher risk to develop MOH was the increasing age (OR 2.2; CI 1.2–4.21; *p* < 0.05) (Figure 4).

### Comparison Between ICHD 3 and ICHD 2 Criteria

According the last version of ICHD, the most frequent diagnosis was CM (67.1%), followed by CTTH (12.2%), NDPH (12.2%), undefined (7.9%), and probable CCTH (pCCTH, 0.5%). Concomitant history of MOH was detected in 41/337 patients (10.8%), among whom 31 suffered from CM and 10 from CTTH.

When we used the ICHD 2, CM was diagnosed in 60.4% of patients, probable CM (pCM) in 11.9%, CCTH in 9.5%, pCCTH in 3.1%, NDPH in 12.2%, and undefined in 2.6%.

**Figure 5:**
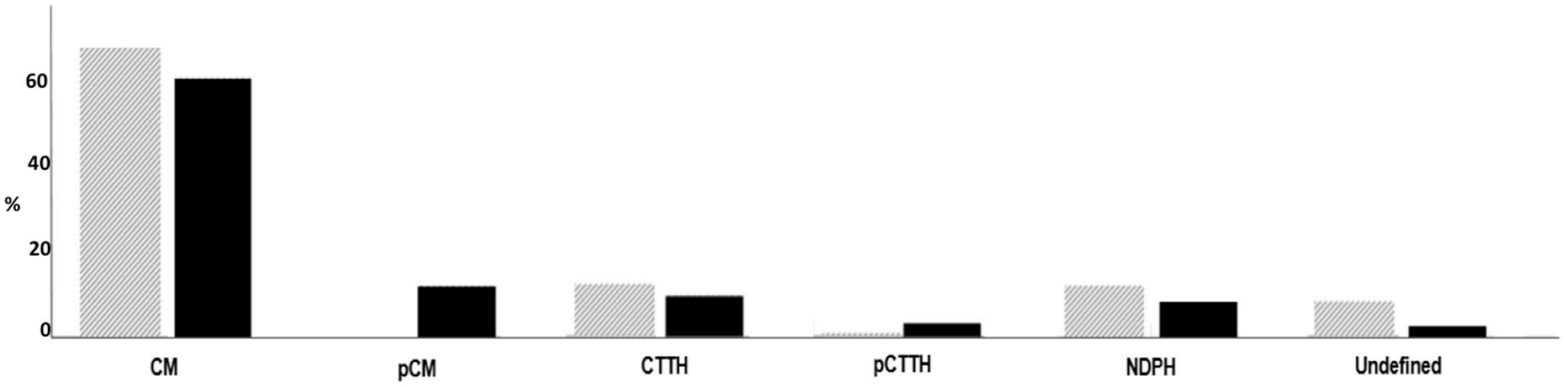
Diagnoses obtained by ICHD 2 (black) and ICHD3 (gray) criteria.

When the diagnoses obtained by ICHD 2 and ICHD 3 were compared, significant differences of frequencies were found for pCM (11.9 vs. 0%; *p* < 0.05) and undefined diagnosis (2.6 vs. 7.9%; *p* < 0.05) (Figure 5). When we considered the total of patients who did not receive a conclusive diagnosis (probable and undefined) we found that for ICHD 2 was 17.6% and ICHD 3 was 8.4% (*p* > 0.05).

### CPH Subtype Predictors

As we have done in the past for episodic primary headache (6), we used a multivariate logistic regression analysis to identify headache features and associated symptoms correlated with a correct diagnosis. We found that the presence of photophobia/phonophobia and nausea/vomiting were significantly associated with the diagnosis of both CM [Odd Ratio (OR) 2.8; confidence interval (CI) 1.76–4.6; positive predictive value (PPV) 81%; *p* < 0.05] and pCM (OR 2.5; CI 1.5–4.1; PPV 78%; *p* < 0.05), whereas it was not associated with the diagnosis of both CCTH (OR 0.17; CI 0.1–0.3; VPP 5%; *p* < 0.05) and pCCTH (OR 0.2; CI 0.1–0.5; VPP 5%; *p* < 0.05).

### Prophylactic Therapy

**Figure 6:**
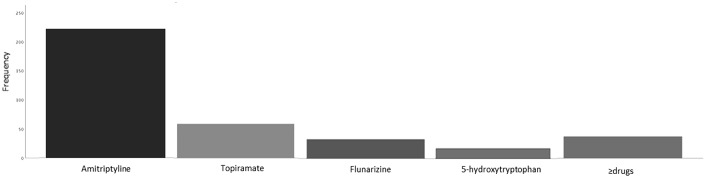
Frequencies of patients that received each drug.

**Figure 7:**
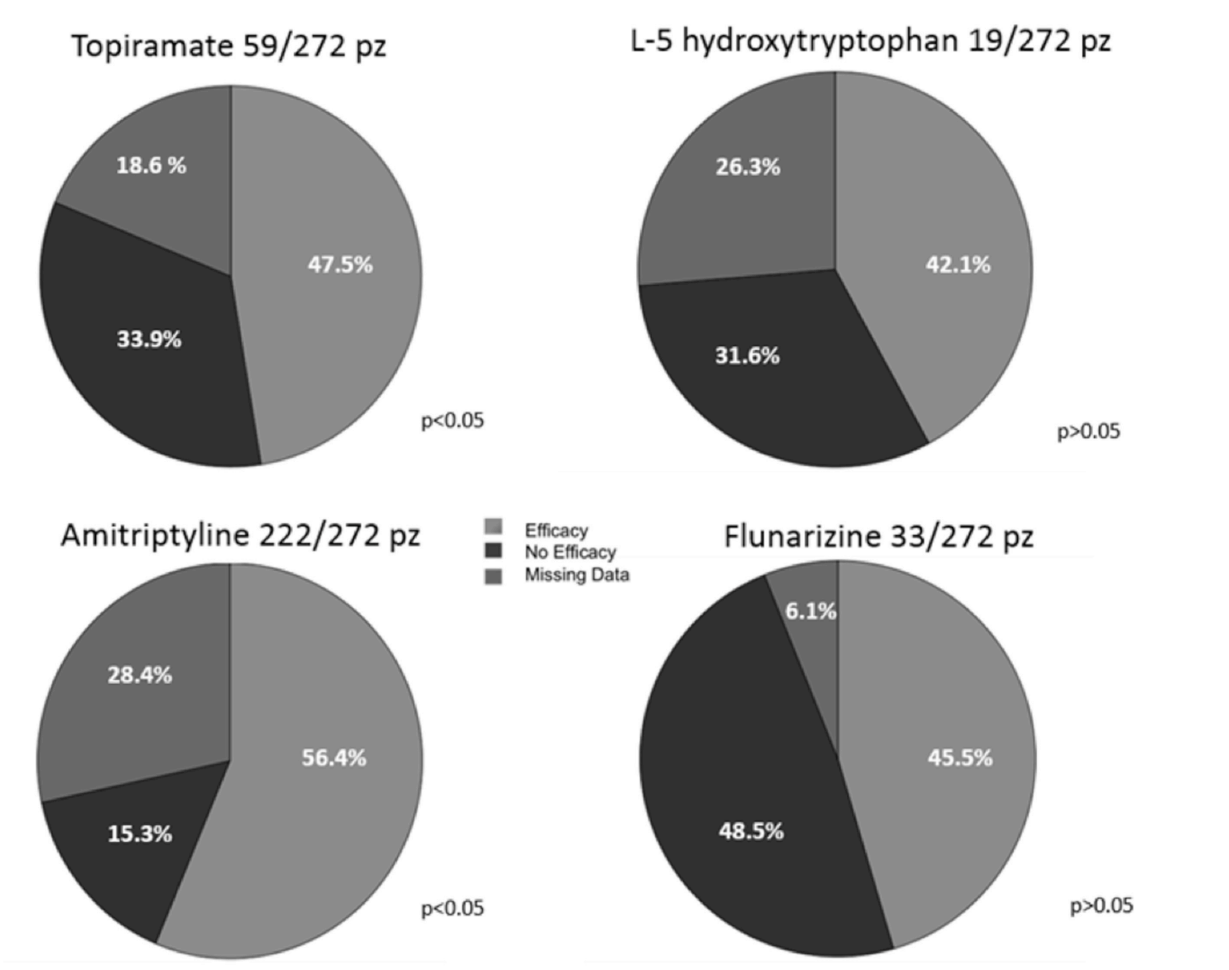
Response to therapy (percentages of patients).

Data concerning the use of prophylactic therapy were issued from 272 patients (72.1%). The drugs used for prophylaxis included 5-hydroxytryptophan, flunarizine, amitriptyline and topiramate. The most frequently used drug was amitriptyline (81.6%), followed by topiramate (21.7%), flunarizine (12%), and 5-hydroxytryptophan (6.9%), while 13.9% of patients needed more than one drug (Figure 6). Around half of patients (54%) had a beneficial response (reduction in the frequency of attacks by at least 50%), while 16.5% of patients showed no improvement. However, we could not have follow-up data for 29.5% of patients. Amitriptyline and topiramate were the drugs with higher percentage of efficacy (*p* < 0.05) and no significant difference in efficacy was found between them (*p* > 0.05) (Figure 7).

## Discussion

CPH represents a growing problem in the pediatric and adolescent age. Our study aimed to fill a lack in the literature regarding CPH description in pediatric age and to verify if the changes made in the third version of ICHD could bring advantages for the diagnosis of CPH in this age group.

The most relevant results of our study were the following:

- ICHD 3 criteria keep presenting limits when applied in pediatric age, especially in children under 6 years of age. The main limit concerns the criterion of the duration of the attack.
- We reported the main correlations between CPH and demographic data and described also the most frequent phenotypes.
- The MOH prevalence in our population was 10.8%, much lower than the in adult patients.
- Amitriptyline and topiramate were the most effective drugs in our CPH patients.

### Has ICHD 3 Given an Advantage?

In our CPH population, female sex was prevalent in the age group between 0 and 6 years and between 15 and 18 years (75% female vs. 24% male in 0–6 years; 81 vs. 19% above 15 years;). Our data confirm the findings of studies on both adult and adolescent chronic headaches which showed an higher frequency in females than males (7–11).

Though largely overlapping, ICHD 2 and ICHD 3 show some differences, especially for the non-conclusive diagnoses. These include the so-called “probable diagnoses” (when one of the criteria is not met) and “undefined diagnoses” (when more than one criterion is not met). While the diagnosis of pCM was possible in the ICHD 2, it was abolished in ICHD 3. Patients defined as pCM with the ICHD 2 belong to two categories: those with simultaneous story of MOH and those who did not meet one of the criteria for diagnosis in particular the duration criterion. According to the ICHD 3, the first ones are re-classified as CM, while the second ones as undefined (2, 5).

According to ICHD 2, in patients overusing medications the diagnosis of MOH can be definitely done only if headache improves after overused medication withdrawal. Before being diagnosed definitely, patients with medication overuse were temporarily given a diagnosis of pCM or pCTTH with probable MOH (5). According to ICHD 3, patients meeting the criteria for CM/CTTH and MOH should be coded for both. After drug withdrawal, headache can either revert to an episodic type or remain chronic, and the patient should be re-diagnosed accordingly (2). In our patients, the modification in MOH diagnosis led to a slight increase of CM prevalence from 60.4% (ICHD 2) to 67.1% (ICHD 3).

While CM can be diagnosed with the ICHD 2 whether the patient refers at least 15 days a month of headache with the clinical characteristics of migraine, the ICHD 3 requires that only eight out of 15 episodes must meet the criteria for migraine. Therefore, we should expect an increase in the CM frequency. However, in our patients the CM prevalence did not largely change passing from the second to the third version of the ICHD. This is probably due to the fact that most our chronic patients with undefined diagnosis did not receive such a diagnosis for the qualitative characteristics of their headache, but for the duration of their attacks. Indeed, we found that most children under 6 years of age (70%) could not satisfy the criterion of the attack duration, often suffering from episodes shorter than 2 h. While the most frequent phenotype in patients over 6 years of age was CM, younger patients showed a significant increase in the prevalence of probable or undefined diagnoses. The associated symptoms were useful for a diagnosis of primary headache (6). In particular, the presence of photophobia and phonophobia was associated with diagnosis of migraine, while the absence of these symptoms was a predictor of CTTH. The problem of the duration of the headache attack confirms our previous data showing that very young children can rarely satisfy the ICHD 3 criteria for the diagnosis of episodic migraine and TTH (6, 12).

Taking into account the whole amount of our patients who cannot receive a conclusive diagnosis (probable and undefined), this percentage dropped from 17.5% with the ICHD 2 criteria to 8.5% with the ICHD 3 criteria. This means that, compared to the ICHD 2, the latest ICHD version shows a higher diagnostic power, even if the criteria for children under 6 years of age need a further improvement.

### Medication Overuse Headache in Pediatric Age

MOH affects 1–2% of the adult general population and 25–50% of the chronic headache population.

This frequency increased to 30–50% if we consider the cephalalgic patients followed in specialized headache centers (13). As for pediatric age, population surveys conducted in Taiwan (14) and Norway (15) found that 0.3 and 0.5% of adolescents respectively could receive a diagnosis of MOH. Considering the population of children and adolescents suffering from headache, there are values of MOH prevalence ranging from 2 to 82.5% (16–20). In our sample, the prevalence of MOH, diagnosed according to the ICHD 3 criteria, was 10.8%. The large variability in MOH can be due to several factors, including differences in genetic background, parenting style, and/or a pediatricians “approach to headache treatment (17).

While in adults MOH is more frequent in CM than in CTTH patients (21), in our young patients there was no significant difference. The main risk factor associated with the development of MOH was the age (OR 2.2; CI 1.2–4.21; *p* < 0.05). Indeed, the proportion of patients with MOH was significantly higher in patients over 15 years than in the other age groups (*p* < 0.05). This finding is conceivable, since adolescents can manage the symptomatic drugs by their own while in younger children the drugs are administered under parental control.

### Pharmacological Treatment of CPH

There are few trials regarding the efficacy of the pharmacological prophylaxis in young headache patients. Evidence of efficacy for prophylactic treatment of episodic migraine in children and adolescents are available for flunarizine, topiramate, and trazodone (unavailable in the USA), topiramate and trazodone (22). The use of amitriptyline, combined with psychological treatment, in patients with CM is supported by one randomized controlled trial (22, 23). However, clear recommendations for the prophylactic treatment of CPH pediatric patients are currently unavailable.

In our population, the most effective drugs were amitriptyline and topiramate without significant differences between them. Mack et al., (24) investigated the efficacy of amitriptyline in patients with high-frequency headache and found that both headache frequency and intensity significantly improved during treatment. They underlined that also chronic daily headaches or continuous headaches appeared to respond to amitriptyline (24). As for topiramate, its efficacy in the prophylaxis of episodic migraine at high frequency has been reported (22, 23), while there are no recommendations for high frequency TTH or chronic headaches. The good efficacy of topiramate in our population suggests that this drug should be considered also for the CPH treatment.

Unfortunately, since follow-up data were missed for a large proportion of our patients, any consistent conclusion about drug effectiveness cannot be drawn. Drop-out patients are mainly those who did not present to the subsequent control visits. There are 3 main possible reasons for drop-outs: (1) some patients, who had improved, did not return to the control visit, (2) other patients, in whom the treatment had not worked, referred to other centers, and (3) adverse events related to drugs. An emerging literature demonstrates that patients with migraine and other headaches hesitate to adhere to pharmacological regimens (25, 26). The lack of adherence to preventive therapies has significant consequences on disease severity, frequency of the attacks and social economics costs. In children and adolescents, the limit of adherence can be improved not only through an accurate education of the patient and his/her parents, but also increasing the evidence about the diagnosis, management and available therapies.

### Limitations of the Study

Our study has some limitations. First, retrospective design of the study can reduce its reliability in the description of the clinical CPH features. However, here we present a picture of pediatric CPH patients referring to third level centers and believe that our data, including those concerning treatment, can be representative also of other similar settings. Second, the findings about the prophylactic treatment are largely affected by the drop-outs and are not placebo controlled. This last point is particularly important, considering the open debate about the efficacy of placebo in children (27–30).

## Conclusions

Literature shows that CPH is a growing phenomenon in the pediatric population. To date, our study includes the most extensive Italian CPH children cohort. We showed that the ICHD3 criteria, though not allowing us to reach a conclusive diagnosis in 8.5% of cases, represented an improvement compared to the ICHD 2 criteria, according to which 17.5% of our patients did not have a definitive diagnosis. The uncertain diagnoses involved 70% of patients under 6 years, being the attack duration, shorter than 2 h, the first cause of uncertainty. MOH prevalence was 10.8% and it was particularly high in patients older than 15 years. Amitriptyline and topiramate proved the most effective drugs, regardless of the headache type.

## Author Contributions

LP is responsible for the design of the study and the writing of the manuscript, supervision of the patients selection and data collection phase, the statistical analysis, and the interpretation of the results. IS participated in the data collection and writing the manuscript. BaB, RM, ST and FD participated in data collection. CT is responsible for data collection from patients of Insubria University Center. BeB participated in data collection of patients from Insubria University Center. PA carried out the statistical analysis. FV contributed to interpretation of results. MV supervised the patients selection and data collection phase, the statistical analysis, and the interpretation of the results.

### Conflict of Interest Statement

The authors declare that the research was conducted in the absence of any commercial or financial relationships that could be construed as a potential conflict of interest.

## References

[1] Lipton RB Manack A Ricci JA Chee E Turkel CC Winner P. Prevalence and burden of chronic migraine in adolescents: results of the chronic daily headache in adolescents study (C-dAS). Headache (2011) 51:693–706. 10.1111/j.1526-4610.2011.01885.x 21521206

[2] Headache classification committee of the international headache society (IHS) The international classification of headache disorders, 3rd edition. Cephalalgia (2018) 38:1–211. 10.1177/0333102413485658 29368949

[3] Kroner JW Hershey AD Kashikar-Zuck SM LeCates SL Allen JR Slater SK. Cognitive behavioral therapy plus amitriptyline for children and adolescents with chronic migraine reduces headache days to ≤ 4 Per Month. Headache (2016) 56:711–6. 10.1111/head.12795 26992129

[4] Arruda MA Chevis CF Bigal ME. Recent advances in the management of chronic migraine in children. Expert Rev Neurother. (2018) 18:231–9. 10.1080/14737175.2018.1438191 29429363

[5] Headache Classification Subcommittee of the International Headache Society (IHS) The international classification of headache disorders, 2nd edition. Cephalalgia (2004) 24:9–160. 10.1111/j.1468-2982.2003.00824.x 14979299

[6] Torriero R Capuano A Mariani R Frusciante R Tarantino S Papetti L. Diagnosis of primary headache in children younger than 6 years: a clinical challenge. Cephalalgia (2017) 37:947–54. 10.1177/0333102416660533 27432612

[7] Eidlitz-Markus T. Zeharia A. Symptoms and clinical parameters of pediatric and adolescent migraine, by gender - a retrospective cohort study. J Headache Pain (2017) 18:80. 10.1186/s10194-017-0789-z 28791575 PMC5548702

[8] Koenig MA Gladstein J McCarter RJ Hershey AD Wasiewski W Pediatric committee of the american headache society. Chronic daily headache in children and adolescents presenting to tertiary headache clinics. Headache (2003) 43:431 10.1046/j.1526-4610.2002.02124.x 12167137

[9] Hershey AD Kabbouche MA Powers SW. Chronic daily headaches in children. Curr Pain Headache Rep. (2006) 10:370–6. 10.1007/s11916-006-0062-7 16945254

[10] Pakalnis A Heyer GL. Seasonal variation in emergency department visits among pediatric headache patients. Headache (2016) 56:1344–7. 10.1111/head.12888 27393745

[11] May A Schulte LH. Chronic migraine: risk factors, mechanisms and treatment. Nat Rev Neurol. (2016) 12:455–64. 10.1038/nrneurol.2016.93 27389092

[12] Balestri M Papetti L Maiorani D Capuano 1 Tarantino S Battan B. Features of aura in paediatric migraine diagnosed using the ICHD 3 beta criteria. Cephalalgia (2017) 38:1742–7. 10.1177/0333102417748571 29239213

[13] Munksgaard SB Jensen RH. Medication overuse headache. Headache (2014) 54:1251–7. 10.1111/head.12408 24990298

[14] Wang SJ Fuh JL Lu SR Juang KD. Chronic daily headache in adolescents: prevalence, impact, and medication overuse. Neurology (2006) 66:193–7. 10.1212/01.wnl.0000183555.54305.fd 16434652

[15] Dyb G Holmen TL Zwart JA. Analgesic overuse among adolescents with headache: the Head-HUNT-Youth Study. Neurology (2006) 66:198–201. 10.1212/01.wnl.0000193630.03650.19 16434653

[16] Wang SJ Fuh JL Lu SR. Chronic daily headache in adolescents: an 8-year follow-up study. Neurology (2009) 73:416–22. 10.1212/WNL.0b013e3181ae2377 19605771

[17] Piazza F Chiappedi M Maffioletti E Galli F Balottin U. Medication overuse headache in school-aged children: more common than expected? Headache (2012) 52:1506–10. 10.1111/j.1526-4610.2012.02221.x 22822711

[18] Cuvellier JC Coutlenier F Joriot-Chekaf S Vallée L. Chronic daily headache in French children and adolescents. Pediatr Neurol. (2008) 38:93–8. 10.1016/j.pediatrneurol.2007.10.001 18206789

[19] Moore AJ Shevell M. Chronic daily headaches in pediatric neurology practice. J Child Neurol. (2004) 19:925–9. 10.1177/08830738040190120301 15704864

[20] Esposito SB Gherpelli JL. Chronic daily headaches in children and adolescents: a study of clinical characteristics. Cephalalgia (2004) 24:476–82. 10.1111/j.1468-2982.2004.00685.x 15154857

[21] Diener HC Holle D Solbach K Gaul C. Medication-overuse headache: risk factors, pathophysiology and management Nat Rev Neurol. (2016) 12:575–83. 10.1038/nrneurol.2016.124 27615418

[22] Arruda MA Bigal ME Headaches and migraines. In: Mahan LFJD editor. Succinct Pediatrics: Evaluation and Management for Common and Critical Care. Elk Grove Village, IL: American Academy of Pediatrics (2015).

[23] Papetti L Spalice A Nicita F Paolino MC Castaldo R Iannetti P. Migraine treatment in developmental age: guidelines update. J Headache Pain (2010) 11:267–76. 10.1007/s10194-010-0205-4 20349201 PMC3451916

[24] Mack KJ. Episodic and chronic migraine in children. Semin Neurol. (2006) 26:223–31. 10.1055/s-2006-939923 16628533

[25] Markus TE Moad B Haimi-Cohen Y Zeharia A. Factors influencing response to pharmacologic treatment of migraine in a pediatric headache clinic. Headache (2016) 56:1120–31. 10.1111/head.12858 27316535

[26] Kroon Van Diest AM Ramsey RR Kashikar-Zuck S Slater S Hommel K Kroner JW. Treatment adherence in child and adolescent chronic migraine patients: results from the cognitive-behavioral therapy and amitriptyline trial. Clin J Pain (2017) 33:892–8. 10.1097/AJP.0000000000000481 28118256 PMC5522369

[27] Kacperski J Bazarsky A. New developments in the prophylactic drug treatment of pediatric migraine: what is new in 2017 and where does it leave us? Curr Pain Headache Rep. (2017) 21:38. 10.1007/s11916-017-0638-4 28779443

[28] Powers SW Coffey CS Chamberlin LA Ecklund DJ Klingner EA Yankey JW CHAMP investigators. trial of amitriptyline, topiramate, and placebo for paediatric migraine. N Engle J Med. (2017) 376:115124 10.1056/NEJMoa1610384 PMC5226887 27788026

[29] Özge A Faedda N Abu-Arafeh I Gelfand AA Goadsby PJ Cuvellier JC. Experts' opinion about the primary headache diagnostic criteria of the ICHD-3rd edition beta in children and adolescents. J Headache Pain (2017) 18:109. 10.1186/s10194-017-0818-y 29285570 PMC5745373

[30] Valeriani M Papetti L Bartoli B Termine C Salfa I Battan B Features of chronic primary headaches (CPH) in children and adolescents referred to two third level headache centers. 11th European Headache Federation Congress jointly with 31st Congress of the Italian Society for the Study of Headaches. J Headache Pain (2017) 18(Suppl 1):P113 10.1186/s10194-017-0817-z PMC5709272 29189948

